# Serum Gamma-Glutamyltransferase Levels Predict Clinical Outcomes in Hemodialysis Patients

**DOI:** 10.1371/journal.pone.0138159

**Published:** 2015-09-16

**Authors:** Woo Yeong Park, Eun Sil Koh, Su-Hyun Kim, Young Ok Kim, Dong Chan Jin, Ho Chul Song, Euy Jin Choi, Yong-Lim Kim, Yon-Su Kim, Shin-Wook Kang, Nam-Ho Kim, Chul Woo Yang, Yong Kyun Kim

**Affiliations:** 1 Department of Internal Medicine, College of Medicine, The Catholic University of Korea, Seoul, Korea; 2 Department of Internal Medicine, College of Medicine, Chung-Ang University, Seoul, Korea; 3 Department of Internal Medicine, Kyungpook National University School of Medicine, Daegu, Korea; 4 Department of Internal Medicine, College of Medicine, Seoul National University, Seoul, Korea; 5 Department of Internal Medicine, College of Medicine, Yonsei University, Seoul, Korea; 6 Department of Internal Medicine, Chonnam National University Medical School, Gwangju, Korea; 7 Cell Death Disease Research Center, The Catholic University of Korea, Seoul, Korea; Kaohsiung Medical University Hospital, Kaohsiung Medical University, TAIWAN

## Abstract

**Background:**

Gamma-glutamyltransferase (GGT) is a biomarker of liver injury. GGT has also been reported to be a marker of oxidative stress and a predictor of mortality in the general population. Hemodialysis (HD) patients suffer from oxidative stress. The aim of our study was to investigate the relationship between serum GGT levels and clinical outcomes in HD patients.

**Methods:**

A total of 1,634 HD patients were enrolled from the Clinical Research Center registry for end-stage renal disease, a prospective cohort in Korea. Patients were categorized into three groups by tertiles of serum GGT levels. The primary outcome was all-cause, cardiovascular, or infection-related mortality and hospitalization.

**Results:**

During the median follow-up period of 30 months, the highest tertile of serum GGT levels had a significantly higher risk for all-cause mortality (hazard ratio (HR) 2.39, 95% confidence interval (CI), 1.55–3.69, P<0.001), cardiovascular mortality (HR 2.14, 95% CI, 1.07–4.26, P = 0.031) and infection-related mortality (HR 3.07, 95% CI, 1.30–7.25, P = 0.011) using tertile 1 as the reference group after adjusting for clinical variables including liver diseases. The highest tertile also had a significantly higher risk for first hospitalization (HR 1.22, 95% CI, 1.00–1.48, P = 0.048) and cardiovascular hospitalization (HR 1.42, 95% CI, 1.06–1.92, P = 0.028).

**Conclusions:**

Our data demonstrate that high serum GGT levels were an independent risk factor for all-cause, cardiovascular, and infection-related mortality, as well as cardiovascular hospitalization in HD patients. These findings suggest that serum GGT levels might be a useful biomarker to predict clinical outcomes in HD patients.

## Introduction

End-stage renal disease (ESRD) patients undergoing hemodialysis (HD) therapy have higher mortality and morbidity compared with the general population [1]. Common causes of mortality in HD patients are cardiovascular and infection-related diseases [2]. Many studies have evaluated biomarkers for predicting clinical outcomes in HD patients. Oxidative stress is one of the useful targets for biomarker because oxidative stress is increased in HD patients and increased oxidative stress is associated with mortality [3].

Gamma-glutamyltransferase (GGT) is a widely used biomarker for liver diseases. In addition, GGT has been known as one of the markers of oxidative stress because it is an enzyme playing an important role in the extracellular catabolism of glutathione (GSH), the representative intracellular antioxidant [4–6]. Oxidative stress mediated by GGT can influence coronary atherosclerotic plaques and endothelial dysfunction [6–8]. Serum GGT levels have been reported to be associated with all-cause mortality and cardiovascular and/or cancer mortality in the general population and in patients with coronary artery disease and type 2 diabetes mellitus [9,10].

In HD patients, there are only few studies on the association between serum GGT levels and clinical outcomes. A previous study reported that high serum GGT levels are associated with all-cause and cardiovascular mortality in HD patients [11]. However, whether GGT is associated with cause-specific mortality and hospitalization remains unclear.

In this study, we investigated the association between serum GGT levels and all-cause, cardiovascular and infection-related mortality in addition to hospitalization from the Clinical Research Center (CRC) registry for ESRD cohort in Korea.

## Patients and Methods

### Study population

This study is a prospective observational cohort study in the CRC registry for ESRD collected from 31 medical centers in Korea. The study period was April 2009-July 2014. The study included adult (> 18 years old) dialysis patients. A total of 3,329 HD patients were enrolled. Patients were excluded if information about serum GGT levels was not available (n = 1,695). A total of 1,634 patients were finally included in this study.

Demographic and clinical data were collected at enrollment. Dates and causes of mortality and hospitalization were reported whenever the events took place.

### Ethics

This study was approved by the institutional review board at each center [The Catholic University of Korea, Bucheon St. Mary's Hospital; The Catholic University of Korea, Incheon St. Mary's Hospital; The Catholic University of Korea, Seoul St. Mary's Hospital; The Catholic University of Korea, St. Mary's Hospital; The Catholic University of Korea, St. Vincent's Hospital; The Catholic University of Korea, Uijeongbu St. Mary's Hospital; Cheju Halla General Hospital; Chonbuk National University Hospital; Chonnam National University Hospital; Chung-Ang University Medical Center; Chungbuk National University Hospital; Chungnam National University Hospital; Dong-A University Medical Center; Ehwa Womens University Medical Center; Fatima Hospital, Daegu; Gachon University Gil Medical Center; Inje University Pusan Paik Hospital; Kyungpook National University Hospital; Kwandong University College of Medicine, Myongji Hospital; National Health Insurance Corporation Ilsan Hospital; National Medical Center; Pusan National University Hospital; Samsung Medical Center, Seoul; Seoul Metropolitan Government, Seoul National University, Boramae Medical Center; Seoul National University Hospital; Seoul National University, Bundang Hospital; Yeungnam University Medical Center; Yonsei University, Severance Hospital; Yonsei University, Gangnam Severance Hospital; Ulsan University Hospital; Wonju Christian Hospital (in alphabetical order)] and performed in accordance to the Declaration of Helsinki. Written informed consent was obtained from all patients.

### Data Collection

Baseline demographic and clinical data including age, gender, height, weight, body mass index (BMI), co-morbidities, laboratory results and therapeutic characteristics were recorded. Serum levels of hemoglobin, albumin, aspartate aminotransferase (AST), alanine aminotransferase (ALT), alkaline phosphatase (ALP), calcium, phosphorus, intact parathyroid hormone (PTH), total cholesterol (TC), triglyceride (TG), uric acid, high-sensitivity C-reactive protein (hsCRP) and ferritin were determined from blood samples. Serum GGT levels were measured at 37°C using fasting blood samples and analyzed by enzyme kinetic assay (Hitachi 7600–210, Tokyo, Japan) using the standard method of the International Federation of Clinical Chemistry (IFCC). In this study, the distribution of GGT levels was skewed to the right (S1 Fig). Thus, patients were categorized into three groups by serum GGT level tertiles as follows: tertile 1, GGT<14.5 IU/L; tertile 2, GGT = 14.5–28 IU/L; tertile 3, GGT>28 IU/L.

### Outcomes

The primary outcomes were all-cause, cardiovascular and infection-related mortality. The secondary outcomes were all-cause, cardiovascular and infection-related hospitalization. All patients were followed until death or the end of the study, with censoring of data at the time that a patient underwent renal transplantation or was lost to follow-up because of patient withdrawal or transfer to a nonparticipating hospital. For each death and hospitalization, the principal investigators of the clinical center completed a form including the cause of death or hospitalization according to the CRC for ESRD study classification.

### Statistical analysis

Data for continuous variables with normal distributions were expressed as the mean ± standard deviation and those without normal distributions were presented as the median with interquartile range. The Student’s t-test, Mann–Whitney test, one-way ANOVA, or Kruskal-Wallis test were used as appropriate to determine differences in continuous variables. Categorical variables are presented as numbers (percentages). The Pearson’s chi-square test or Fisher’s exact test were used to compare differences in categorical variables.

Absolute mortality rates were calculated per 100 person-years of follow-up. Patient survival rates were calculated by the Kaplan-Meier method using the log-rank test. The Cox proportional hazard regression model was used to calculate the hazard ratio (HR) and 95% confidence interval (CI) for all-cause, cardiovascular and infection-related mortality as well as hospitalization using tertile 1 as the reference category. The assumption of proportional hazards over time was assessed by visual inspection of a log-minus-log survival plot. The Cox models were adjusted for significant or nearly significant (p < 0.1) predictor for all-cause mortality in univariate Cox regression analysis including age, diabetes mellitus, cardiovascular diseases, hemoglobin levels, serum levels of ferritin, albumin, ALP and HCV Ab. To have adequate confounder control, important covariates known to be influential based on prior studies and clinical insight, such as gender, serum levels of AST, hs-CRP, TC and HBs Ag were retained in the multivariate Cox regression model regardless of their statistical significance. Analyses were adjusted for potential confounders using two models. Model 1 was adjusted for age and gender. Model 2 was adjusted for age, gender, diabetes mellitus, cardiovascular diseases, hemoglobin levels, serum levels of ferritin, serum hs-CRP, albumin, AST, ALT, ALP, TC, HBs Ag and HCV Ab.

A value of P<0.05 was considered statistically significant. All statistical analyses were performed using SPSS 16.0 software (Chicago, IL, USA).

## Results

### Patient characteristics

The median GGT level was 20 IU/L (interquartile range: 12–35 IU/L). Baseline characteristics of the study population by serum GGT level tertile are shown in Table 1. Patients with higher GGT levels were older and a higher proportion of patients were male. Among the causes of ESRD, diabetes mellitus was more common in patients with higher GGT levels than in those with lower GGT levels. Patients with high GGT levels had a higher prevalence of diabetes mellitus, anti-HCV Ab positivity, had higher serum levels of AST, ALT, ALP, ferritin and hsCRP, and had lower diastolic blood pressure and lower serum levels of phosphorous and intact PTH. There were no significant differences in BMI, prevalence of cardiovascular diseases, duration of dialysis therapy, systolic blood pressure, hemoglobin levels, serum levels of TC, TG and uric acid among the groups.

**Table 1:** Baseline characteristics of the study population by serum GGT tertiles.

Characteristics		GGT (IU/L)			P
	Overall	Tertile 1	Tertile 2	Tertile 3	
		< 14.5	14.5–28	> 28	
**Number of patients**	1,634	547	566	521	
**GGT (IU/L)**	20 (12–35)	11 (9–12)	20 (17–25)	51 (37–88)	< 0.001
**Age (years)**	58 ± 14	57 ± 15	59 ± 13	59 ± 12	0.001
**Male, n (%)**	1,001 (61.3)	276 (50.5)	355 (62.7)	370 (71.0)	< 0.001
**Body mass index (kg/m** ^**2**^ **)**	22.7 ± 3.5	22.6 ± 3.3	22.6 ± 3.5	22.7 ± 3.6	0.950
**Primary renal disease, n (%)**					0.006
**Diabetes mellitus**	840 (51.6)	270 (49.4)	286 (50.8)	284 (54.8)	
**Glomerulonephritis**	220 (13.5)	78 (14.3)	76 (13.5)	66 (12.7)	
**Polycystic kidney disease**	40 (2.5)	13 (2.4)	4 (0.7)	23 (4.4)	
**Others**	528 (32.4)	186 (34.0)	197 (35.1)	145 (28.0)	
**Co-morbidity, n (%)**					
**Diabetes mellitus**	876 (55.9)	271 (51.9)	305 (56.2)	300 (59.8)	0.040
**Cardiovascular diseases**	499 (32.0)	151 (29.1)	176 (32.7)	172 (34.3)	0.186
**Duration of dialysis therapy, months**	19 ± 41	19 ± 43	18 ± 38	20 ± 42	0.743
**Systolic BP (mmHg)**	143 ± 24	143 ± 23	143 ± 24	142 ± 25	0.841
**Diastolic BP (mmHg)**	77 ± 14	78 ± 14	78 ± 13	76 ± 15	0.005
**Hemoglobin (g/dl)**	9.4 ± 1.8	9.4 ± 1.7	9.4 ± 1.8	9.4 ± 1.8	0.710
**Serum AST (IU/L)**	24 ± 48	18 ± 21	21 ± 43	33 ± 68	< 0.001
**Serum ALT (IU/L)**	22 ± 58	14 ± 13	19 ± 39	34 ± 92	< 0.001
**Serum ALP (IU/L)**	82 (62–121)	74 (55–109)	78 (61–108)	97 (72–152)	< 0.001
**Serum albumin (g/dl)**	3.5 ± 0.6	3.6 ± 0.6	3.6 ± 0.6	3.5 ± 0.6	0.038
**Serum calcium (mg/dl)**	8.3 ± 4.0	8.0 ± 1.3	8.6 ± 6.1	8.3 ± 3.0	0.002
**Serum phosphorus (mg/dl)**	5.3 ± 1.9	5.5 ± 2.1	5.3 ± 1.9	5.1 ± 1.7	0.001
**Serum intact PTH (pg/ml)**	176 (91–311)	193 (88–334)	186 (94–320)	160 (87–277)	0.001
**Serum TC (mg/dl)**	153 ± 44	153 ± 44	155 ± 45	150 ± 44	0.137
**Serum TG (mg/dl)**	122 ± 75	120 ± 64	122 ± 74	122 ± 86	0.850
**Serum uric acid (mg/dl)**	8.0 ± 2.4	8.1 ± 2.3	8.0 ± 2.4	7.8 ± 2.4	0.236
**Serum hsCRP (mg/dl)**	0.4 (0.1–1.8)	0.3 (0.1–1.2)	0.4 (0.1–1.8)	0.6 (0.1–2.5)	0.013
**Serum Ferritin (ng/ml)**	222 (116–405)	203 (103–355)	221 (120–387)	253 (123–490)	< 0.001
**HBs Ag (+), n (%)**	99 (6.1)	27 (4.9)	32 (5.7)	40 (7.7)	0.127
**HCV Ab (+), n (%)**	49 (3.0)	12 (2.2)	13 (2.3)	24 (4.6)	0.009

Values for continuous variables given as means ± standard deviation and variables not normally distributed given as median and interquartile range; values for categorical variables given as numbers (percentages). GGT, gamma-glutamyltransferase; BP, blood pressure; AST, aspartate aminotransferase; ALT, alanine aminotransferase; ALP: alkaline phosphatase; PTH, parathyroid hormone; TC, total cholesterol; TG, triglyceride; hsCRP, high-sensitivity C-reactive protein; HBs Ag, hepatitis B surface antigen; HCV Ab, hepatitis C virus antibody.

### Determinants of serum GGT levels

Serum GGT levels were positively correlated with age, serum levels of AST, ALT, ALP, hsCRP and ferritin and negatively correlated with diastolic BP and serum levels of albumin, phosphorus and intact PTH (S1 Table). In stepwise multiple linear regression model including all univariate correlates of GGT in HD patients, male sex (β = 0.11, P<0.001), serum AST (β = 0.10, P<0.001), serum ALP (β = 0.49, P<0.001), serum TC (β = 0.07, P = 0.004) and serum ferritin (β = 0.13, P<0.001) levels were independently correlated with serum GGT levels.

### Association between serum GGT levels and all-cause, cardiovascular and infection-related mortality

The median follow-up period was 30 months (interquartile range: 12–47 months). During the follow-up period, 712 patients left the study. The reasons for censoring included kidney transplantation (n = 104), transfer to a nonparticipating hospital (n = 225), refusal of further participation (n = 107) or others (n = 112). There were 164 deaths during the follow-up period.

Cardiovascular disease was the leading cause of death (37.8% of all deaths), followed by infection-related disease (27.4% of all deaths).

**Fig 1:**
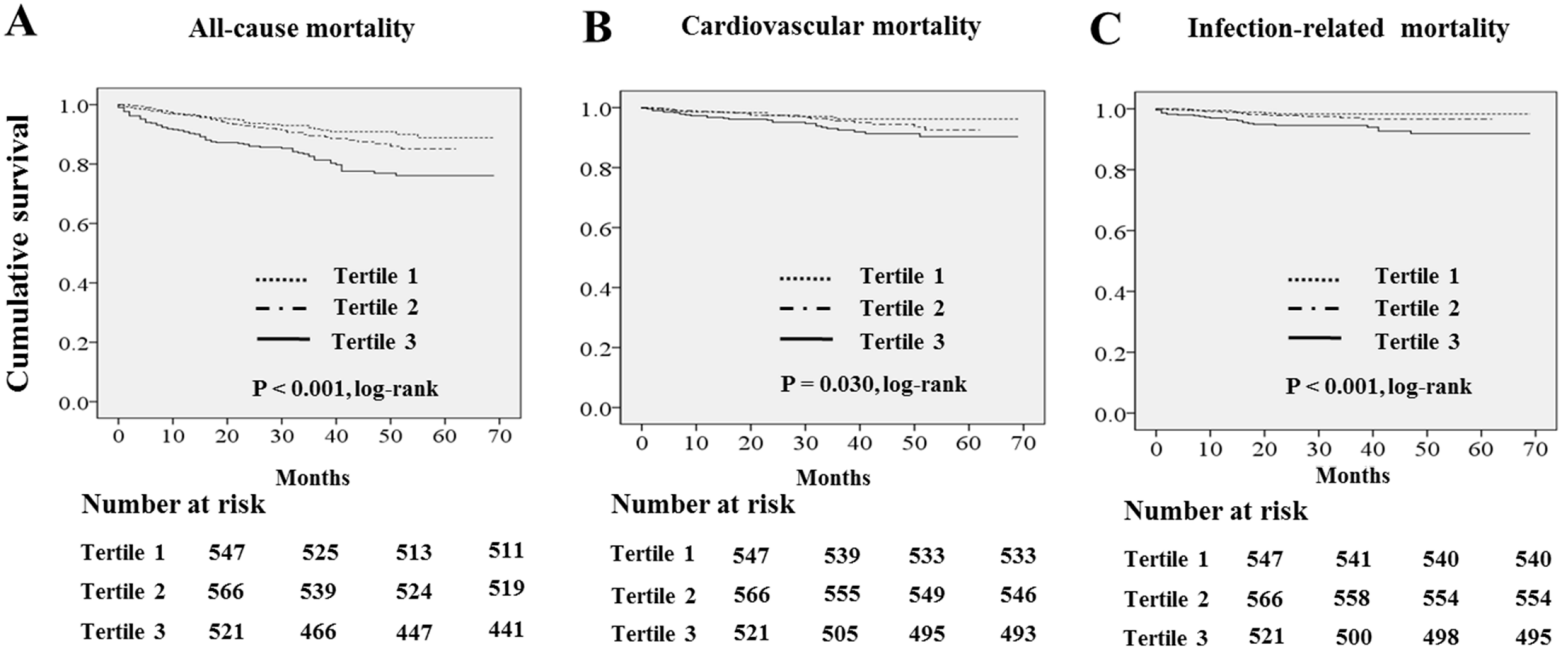
Kaplan-Meier survival curve for all-cause, cardiovascular, infection-related mortality according to serum GGT tertiles.

The absolute mortality rate was 4.3 deaths per 100 person-years. Fig 1 show the Kaplan-Meier plot of patient survival according to tertiles of serum GGT levels. The log rank test showed that all-cause, cardiovascular, and infection-related mortality rates were significantly increased in patients in the highest serum GGT tertile (P<0.001, P = 0.030, P<0.001, respectively).

Univariate and multivariate Cox regression analyses for all-cause, cardiovascular and infection-related mortality are shown in Table 2. In the crude model, the HRs for all-cause, cardiovascular, and infection-related mortality of patients in tertile 3 of serum GGT levels were 2.51 (95% CI, 1.69–3.72, P<0.001), 2.25 (95% CI, 1.19–4.28, P = 0.013) and 4.20 (95% CI, 1.82–9.68, P = 0.001) respectively, using tertile 1 as the reference category. In multivariate Cox regression analysis, tertile 3 had a significantly higher risk for all-cause mortality than tertile 1 even after adjusting for demographics, laboratory data, and comorbid conditions (model 1: HR 2.40, 95% CI, 1.62–3.56, P<0.001; model 2: HR 2.39, 95% CI, 1.55–3.69, P<0.001). Tertile 3 also was at higher risk for cardiovascular and infection-related mortality in model 1 (HR 2.16, 95% CI, 1.14–4.10, P = 0.019, and HR 4.08, 95% CI, 1.77–9.40, P = 0.001, respectively) and model 2 (HR 2.14, 95% CI, 1.07–4.26, P = 0.031 and HR 3.07, 95% CI, 1.30–7.25, P = 0.011, respectively).

**Table 2:** Univariate and multivariate Cox regression analyses for all-cause, cardiovascular and infection-related mortality.

	Crude model			Model 1			Model 2		
	HR	95% CI	P	HR	95% CI	P	HR	95% CI	P
**All-cause mortality**									
**Tertile 1**		1 (reference)			1 (reference)			1(reference)	
**Tertile 2**	1.29	0.83–1.99	0.254	1.14	0.74–1.76	0.558	1.16	0.71–1.88	0.558
**Tertile 3**	2.51	1.69–3.72	< 0.001	2.40	1.62–3.56	< 0.001	2.39	1.55–3.69	< 0.001
**Cardiovascular mortality**									
**Tertile 1**		1 (reference)			1 (reference)			1(reference)	
**Tertile 2**	1.41	0.71–2.78	0.328	1.24	0.63–2.46	0.533	1.10	0.52–2.36	0.799
**Tertile 3**	2.25	1.19–4.28	0.013	2.16	1.14–4.10	0.019	2.14	1.07–4.26	0.031
**Infection-related mortality**									
**Tertile 1**		1 (reference)			1 (reference)			1 (reference)	
**Tertile 2**	1.69	0.66–4.28	0.272	1.49	0.59–3.77	0.406	1.28	0.49–3.36	0.621
**Tertile 3**	4.20	1.82–9.68	0.001	4.08	1.77–9.40	0.001	3.07	1.30–7.25	0.011

Model 1: Multivariate model including age and gender

Model 2: Multivariate model including model 1 + diabetes mellitus, cardiovascular diseases, hemoglobin levels, serum ferritin levels, serum hs-CRP levels, serum albumin levels, serum AST levels, serum ALT levels, Serum ALP levels, serum cholesterol levels, HBs Ag and HCV Ab.

GGT, gamma-glutamyltransferase; HR, hazard ratio; CI, confidence interval.

### Association between serum GGT level and first, cardiovascular and infection-related hospitalization

A total of 790 hospitalization events occurred during the follow-up period. The most common causes of hospitalization were cardiovascular-related (n = 303, 18.5%) and infection-related hospitalizations (n = 186, 11.4%). Table 3 shows the distribution of causative diseases in patients with cardiovascular and infection-related hospitalization during the follow-up period. Ischemic heart disease and vascular access thrombosis were the most common causes of cardiovascular hospitalization. There were no significant differences in the distribution of causative disease in patients with cardiovascular hospitalization according to serum GGT level tertiles (P = 0.533). Respiratory infection was the most common cause of infection-related hospitalization, and the distribution of causative diseases in patients with infection-related hospitalization was not different among the tertiles of serum GGT levels (P = 0.597).

**Table 3:** Distribution of causative diseases in patients with cardiovascular and infection-related hospitalization during the follow-up period.

	GGT (U/L)			
	Tertile 1	Tertile 2	Tertile 3	P
**Cardiovascular hospitalization**				0.533
**Ischemic heart diseases, n (%)**	13 (15.3)	29 (26.6)	21 (19.3)	
**Congestive heart failure, n (%)**	12 (14.1)	11 (10.1)	16 (13.8)	
**Arrhythmia, n (%)**	7 (8.2)	6 (5.5)	5 (4.6)	
**Cerebral vascular diseases, n (%)**	8 (9.4)	13 (11.9)	13 (11.9)	
**Peripheral vascular diseases, n (%)**	2 (2.4)	1 (0.9)	5 (4.6)	
**Vascular access thrombosis**	39 (45.9)	39 (35.8)	41 (37.6)	
**Other cardiovascular diseases, n (%)**	4 (4.7)	10 (9.2)	9 (8.3)	
**Total, n (%)**	85 (100)	109 (100)	109 (100)	
**Infection-related hospitalization**				0.597
**Respiratory infection, n (%)**	26 (44.1)	26 (52.0)	35 (45.5)	
**Gastrointestinal infection, n (%)**	8 (13.6)	3 (6.0)	7 (9.1)	
**Urinary tract infection, n (%)**	3 (5.1)	1 (2.0)	8 (10.4)	
**Musculoskeletal and soft tissue infection, n (%)**	13 (22.0)	9 (18.0)	16 (20.8)	
**Bacteremia (unknown cause), n (%)**	4 (6.8)	3 (6.0)	2 (2.6)	
**Vascular access related infection**	2 (3.4)	1 (2.0)	1 (1.3)	
**Other infections, n (%)**	3 (5.1)	7 (14.0)	8 (10.4)	
**Total, n (%)**	59(100)	50 (100)	77 (100)	

GGT, gamma-glutamyltransferase.

**Fig 2:**
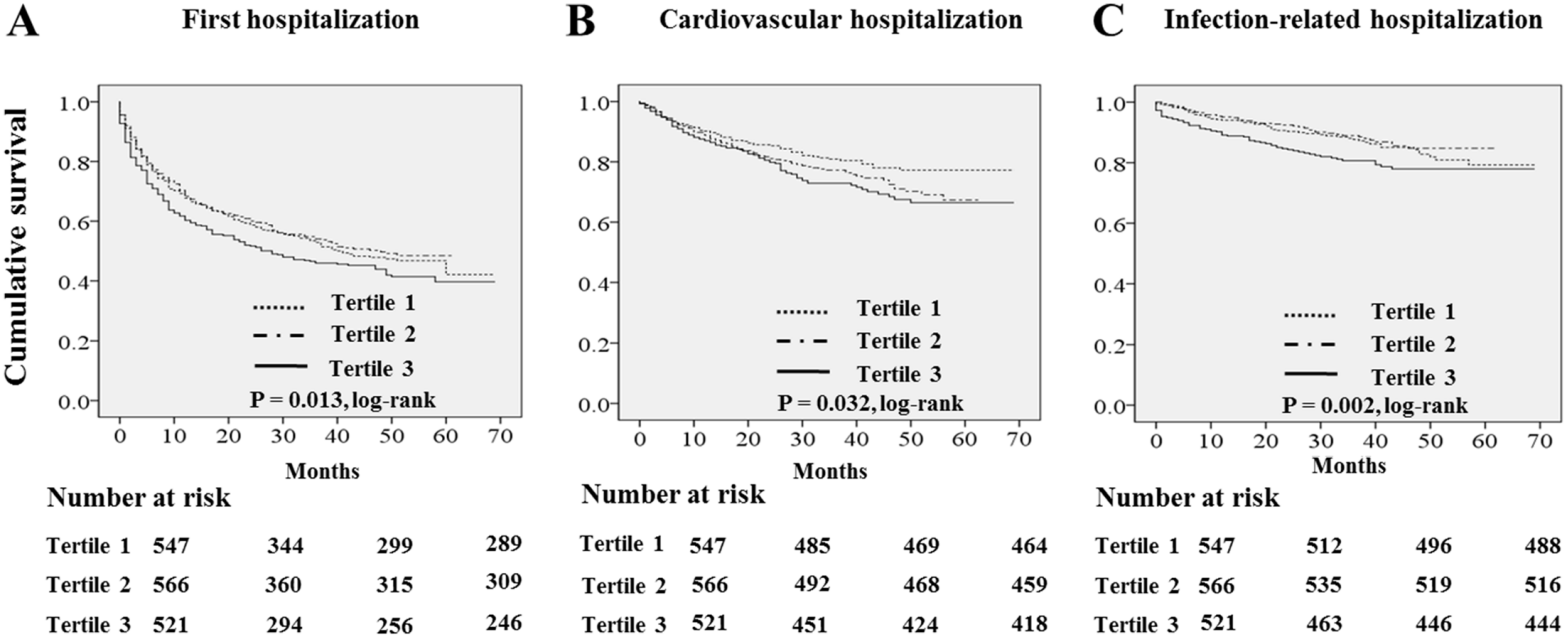
Kaplan-Meier survival curve for all-cause, cardiovascular, infection-related hospitalization according to serum GGT tertiles.

Fig 2 show the Kaplan-Meier plot of hospitalization according to tertiles of serum GGT levels. The log rank test showed that first, cardiovascular, and infection-related hospitalization rate were significantly increased in patients with the highest tertile of serum GGT levels (P = 0.013, P = 0.032 and P = 0.002, respectively). Table 4 shows the univariate and multivariate Cox regression analysis for first, cardiovascular, and infection-related hospitalization. In the crude model, the HRs for first, cardiovascular, and infection-related hospitalization of patients in tertile 3 of serum GGT levels were 1.21 (95% CI, 1.02–1.43, P = 0.029), 1.47 (95% CI, 1.10–1.96, P = 0.010) and 1.53 (95% CI, 1.08–2.14, P = 0.015), respectively, using tertile 1 as the reference category. In multivariate Cox regression analysis, tertile 3 had a significantly higher risk for first hospitalization (model 1: HR 1.23, 95% CI, 1.04–1.47, P = 0.018; model 2: HR 1.22, 95% CI, 1.00–1.48, P = 0.048). Tertile 3 also was at higher risk for cardiovascular hospitalization in model 1 (HR 1.40, 95% CI, 1.05–1.88, P = 0.020) and model 2 (HR 1.42, 95% CI, 1.06–1.92, P = 0.028). Tertile 3 was associated with infection-related hospitalization in model 2 (HR 1.47, 95% CI, 1.04–2.06, P = 0.027). However, its significance was attenuated in model 3 (HR 1.42, 95% CI, 0.99–2.04, P = 0.060).

**Table 4:** Univariate and multivariate Cox regression analyses for all-cause, cardiovascular and infection-related hospitalization.

	Crude model			Model 1			Model 2		
	HR	95% CI	P	HR	95% CI	P	HR	95% CI	P
**First hospitalization**									
**Tertile 1**		1 (reference)			1 (reference)			1 (reference)	
**Tertile 2**	0.95	0.80–1.13	0.592	0.95	0.80–1.14	0.595	0.90	0.74–1.09	0.287
**Tertile 3**	1.21	1.02–1.43	0.029	1.23	1.04–1.47	0.018	1.22	1.00–1.48	0.048
**Cardiovascular hospitalization**									
**Tertile 1**		1 (reference)			1 (reference)			1 (reference)	
**Tertile 2**	1.28	0.96–1.71	0.089	1.20	0.90–1.60	0.206	1.22	0.89–1.66	0.216
**Tertile 3**	1.47	1.10–1.96	0.010	1.40	1.05–1.88	0.021	1.42	1.06–1.92	0.028
**Infection-related hospitalization**									
**Tertile 1**		1 (reference)			1 (reference)			1 (reference)	
**Tertile 2**	0.83	0.57–1.21	0.334	0.79	0.54–1.16	0.227	0.82	0.55–1.23	0.337
**Tertile 3**	1.53	1.08–2.14	0.015	1.47	1.04–2.06	0.027	1.42	0.99–2.04	0.060

Model 1: Multivariate model including age and gender. Model 2: Multivariate model including model 1 + diabetes mellitus, cardiovascular diseases, hemoglobin levels, serum ferritin levels, serum hs-CRP levels, serum albumin levels, serum AST levels, serum ALT levels, Serum ALP levels, serum cholesterol levels, HBs Ag and HCV Ab. GGT, gamma-glutamyltransferase; HR, hazard ratio; CI, confidence interval.

### Association between serum GGT level and liver diseases-related mortality or hospitalization

A total of 7 liver diseases-related death and 14 liver diseases-related hospitalization events occurred during the follow-up period. Most cause of liver diseases-related death was hepatic failure and most causes of liver diseases-related hospitalization were complications of liver cirrhosis including uncontrolled ascites or varix bleeding. Kaplan-Meier plot of patient survival of liver diseases-related mortality or hospitalization according to tertiles of serum GGT levels. The log rank test showed that liver diseases-related clinical outcomes were significantly increased in patients in the highest serum GGT tertile (P<0.001) (S2 Fig).

Univariate and multivariate Cox regression analyses for of liver diseases-related mortality or hospitalization are shown in Table 5. In the univariate analysis, the HR for liver diseases-related clinical outcomes of patients in tertile 3 of serum GGT levels were 15.74 (95% CI, 2.07–119.68, P = 0.008) using tertile 1 as the reference category. In multivariate Cox regression analysis, tertile 3 had a significantly higher risk for liver diseases-related clinical outcomes even after adjusting for age, gender, diabetes mellitus, cardiovascular diseases, serum albumin levels, serum AST levels, serum ALT levels, HBs Ag and HCV Ab (HR 15.110, 95% CI, 1.91–119.71, P = 0.010).

**Table 5:** Univariate and multivariate Cox regression analyses for liver diseases-related mortality or hospitalization.

		Univariate model			Multivariate model		
		HR	95% CI	P	HR	95% CI	P
**Liver diseases-related mortality or hospitalization**							
	Tertile 1		1 (reference)			1(reference)	
	Tertile 2	2.93	0.31–28.2	0.351	2.14	0.19–23.84	0.536
	Tertile 3	15.74	2.07–119.68	0.008	15.11	1.91–119.71	0.010

Multivariate model including age, gender, diabetes mellitus, cardiovascular diseases, serum albumin levels, serum AST levels, serum ALT levels, HBs Ag and HCV Ab.

GGT, gamma-glutamyltransferase; HR, hazard ratio; CI, confidence interval.

## Discussion

In this study, we demonstrated that higher serum GGT levels were significantly associated with increased risk of all-cause, cardiovascular and infection-related mortality. Moreover, higher serum GGT levels were also associated with hospitalization after adjustment for a range of potential confounding factors and liver function in HD patients. These findings suggest that serum GGT levels have prognostic importance and may be an independent predictor for clinical outcomes in HD patients.

GGT is widely used as a biomarker for excessive alcohol consumption and liver diseases in clinical practice [12]. GGT also plays a fundamental role in oxidative stress mechanisms and serum GGT levels are strongly associated with cardiovascular risk factors and diseases in the general population as well as in patient with coronary artery disease or type 2 diabetes mellitus [10].

This study was performed in a HD population, whose oxidative stress is increased compared with the general population because of increased pro-oxidant activity related to old age, high prevalence of diabetes mellitus and hypertension, inflammation, and incompatibility of dialysis membranes [13,14]. Data on the association between serum GGT levels and clinical outcomes in a HD population are limited. One previous study reported that serum GGT levels have the prognostic power to predict all-cause and cardiovascular mortality in HD patients, which is consistent with the results of our study [15].

Our study has some interesting findings First, high serum GGT levels predicted not only cardiovascular mortality but also infection-related mortality as a non-cardiovascular mortality in HD patients. Indeed, numerous large cohort studies have demonstrated an association between GGT activity and non-cardiovascular mortality, particularly cancer, diabetes, and liver disease-related mortality [16–22]. However, the association between serum GGT levels and infection-related mortality has not been established. Our findings support the speculation that elevated GGT levels can affect non-cardiovascular mortality. Furthermore, because infection-related mortality is one of the most common causes of death in HD patients, our findings provide the prognostic power of serum GGT level to predict non-cardiovascular clinical outcomes, especially infection-related mortality, in HD patients. The mechanism underlying the relationship between GGT levels and infection-related mortality is uncertain. However, considering the possibility that elevated levels of reactive oxygen species during infection may have fatal consequences [23], the relationship between GGT levels and oxidative stress may be a potential explanation for the association between GGT levels and infection-related mortality. Further investigation of the association between GGT levels and infection-related clinical outcomes is needed.

Second, our study showed that the association between serum GGT levels and clinical outcomes was independent of liver function and liver diseases. Liver diseases such as hepatitis B and hepatitis C are prevalent in HD patients and hepatitis C has been reported to be a risk factor for mortality and morbidity [24]. Considering that serum GGT levels are a biomarker of liver function and liver diseases, we performed analyses that were adjusted for liver disease, including HBS Ag and anti-HCV Ab positivity, as well as for liver function, including serum AST and ALT levels, to determine whether the association of serum GGT levels is dependent or independent of liver disease or liver function. In this study, serum GGT levels were associated with clinical outcomes even after adjustment for liver disease and liver function.

In addition to adjustment for liver diseases, to clarify the prognostic value of serum GGT levels independent from liver diseases, we excluded the patient with HBV positivity (n = 99), HCV positivity (n = 49) or serum ALT levels > 40 IU/L (n = 114) from enrolled patients and performed survival analyses [25]. The prognostic significance of serum GGT levels still remained even when the patients with liver diseases were excluded in the study cohort. In the crude model, the HRs for all-cause mortality of patients in tertile 3 of serum GGT levels were 2.29 (95% CI, 1.51–3.49, P<0.001) using tertile 1 as the reference category. In multivariate Cox regression analysis, tertile 3 had a significantly higher risk for all-cause mortality than tertile 1 (model 1: HR 2.28, 95% CI, 1.48–3.51, P<0.001; model 2: HR 1.95, 95% CI, 1.17–3.24, P = 0.011). Our data showed that serum GGT levels had prognostic value for clinical outcomes independent from liver disease and liver function.

Third, our study showed that higher serum GGT levels were associated with higher hospitalization rates as well as mortality rate in HD patients. Hospitalization rates in HD patients are markedly increased compared with the general population [26]. Furthermore, hospitalization is associated with a high rate of rehospitalization, emergency department visits and/or death after hospital discharge in HD patients [26,27]. Therefore, biomarkers to predict hospitalization may be helpful to detect HD patients with a high risk of hospitalization and provide careful attention for them. The results of our study contribute to the prognostic power of GGT levels for clinical outcomes in HD patients.

Fourth, our study showed that higher serum GGT levels were associated with higher risk of liver diseases-related clinical outcomes in HD patients. It has been reported that serum GGT levels were associated with hepatocellular carcinoma in HBV or HCV patients [28,29]. The prevalence of hepatitis B or hepatitis C is high in HD patients [24]. However, it is unclear whether serum GGT levels are associated with liver diseases-related mortality or hospitalization in HD patients. We investigated the prognostic value of serum GGT levels to predict liver diseases-related mortality or hospitalization in this study. Although the incidence rate of liver diseases-related death or hospitalization event was relatively lower than those of cardiovascular or infection-related clinical events, higher serum GGT levels had independent association with liver diseases-related mortality or hospitalization. These finding provided the evidence of impact of serum GGT as a traditional biomarker to predict liver diseases in the population of HD treatment.

What is the optimal cut-off value to predict all-cause mortality? We performed the area under the receiver-operating characteristic curve (AUROC) analysis. Serum GGT levels were modestly predictive of all-cause mortality (AUROC, 0.61; 95% CI, 0.57–0.66, P<0.001). The optimal cut-off value using Youden index [30] (sensitivity+specificity-1) was 29.5 IU/L (sensitivity 50%, specificity 71%), which is similar with the lower limit of tertile 3 of serum GGT levels (28 IU/L) in this study. The prognostic significance of serum GGT levels still remained when the patients were divided by the cut-off value of 29.5 IU/L. The HR for all-cause mortality in patients with serum GGT levels ≥ 29.5 IU/L was 2.13 (95% CI 1.45–2.93, P<0.001) using group with serum GGT levels < 29.5 IU/L as the reference category after adjustment for age, gender, diabetes mellitus, cardiovascular diseases, serum albumin levels, serum AST levels, serum ALT levels, HBs Ag and HCV Ab.

Serum levels of liver enzymes such as AST or ALT were lower in patients with HD than those with normal renal function [31]. The reason for this reduction may be due to hemodilution by blood sampling just before HD session, lower serum pyridoxine levels or higher homocysteine levels [31]. There are limited data on comparing the serum GGT levels between HD patients and general population. In this study, the median value of serum GGT levels was 20 IU/L, which is comparable with those of studies including non-dialysis patients (17–31 IU/L) [8–10,16–22]. Furthermore, the reported cut-off value of serum GGT levels predicting clinical outcomes in previous studies in including non-dialysis patients raged from 17–36 IU/L [8–10,16–22], which is also comparable with cut-off value of this study (29.5 IU/L). We cautiously suggest that the prognostic impact of serum GGT levels on clinical outcomes may not different between HD patients and non-dialysis patients.

Our study has some limitations. First, alcohol consumption was not measured in this study. Serum GGT levels are affected by alcohol intake; therefore, we could not examine confounding factors caused by alcohol consumption. However, serum AST, ALT, ferritin and albumin levels were reported to predict alcohol consumption even rather low levels of alcohol intake [32]. In consideration that serum GGT levels predicted clinical outcomes independently from serum AST, ALT, ferritin and albumin levels in this study, serum GGT levels may have prognostic value independent from alcohol consumption in HD patients. Second, some previous studies reported that serum GGT levels are associated with cancer mortality [16]. In this study, deaths from cancer were too infrequent to analyze the association between GGT levels and cancer mortality.

In conclusion, our study demonstrated that high serum GGT levels are an independent risk factor for all-cause mortality and hospitalization in HD patients. Moreover, analysis of the association between serum GGT levels and cause-specific mortality showed that high serum GGT levels were significantly associated with not only cardiovascular mortality, but also infection-related mortality in HD patients. Our findings suggest that serum GGT levels might be a useful biomarker for predicting clinical outcomes in HD patients.

## Supporting Information

S1 Table Spearman correlation of serum GGT levels and clinical parameters. (DOCX)

S1 Fig The distribution of serum GGT levels in enrolled patients. (TIF)

S2 Fig Kaplan-Meier survival curve for liver diseases-related mortality or hospitalization according to serum GGT tertiles. (TIF)
