# Supplementary material for: Serum Gamma-Glutamyltransferase Levels Predict Clinical Outcomes in Hemodialysis Patients
Source: PLoS One. 2015 Sep 16;10(9):e0138159. doi: 10.1371/journal.pone.0138159 (PMC4573328; doi:10.1371/journal.pone.0138159)
Supplement: S1 Table — (DOCX) [file pone.0138159.s001.docx]

|  | r | *P* |
| --- | --- | --- |
| Age (years) | 0.075 | 0.003 |
| Body mass index (kg/m^2^) | 0.003 | 0.916 |
| Duration of dialysis, (months) | -0.018 | 0.460 |
| Systolic BP (mmHg) | -0.023 | 0.363 |
| Diastolic BP (mmHg) | -0.087 | 0.001 |
| Hemoglobin (g/dl) | -0.028 | 0.259 |
| Serum AST (IU/L) | 0.360 | <0.001 |
| Serum ALT (IU/L) | 0.406 | <0.001 |
| Serum ALP (IU/L) | 0.223 | <0.001 |
| Serum hsCRP (mg/dl) | 0.115 | <0.001 |
| Serum Ferritin (ng/ml) | 0.123 | <0.001 |
| Serum albumin (g/dl) | -0.076 | 0.002 |
| Serum calcium (mg/dl) | 0.024 | 0.340 |
| Serum phosphorus (mg/dl) | -0.080 | 0.001 |
| Serum intact PTH (pg/ml) | -0.060 | 0.019 |
| Serum TC (mg/dl) | -0.035 | 0.163 |
| Serum TG (mg/dl) | -0.049 | 0.061 |
| Serum uric acid (mg/dl) | 0.042 | 0.094 |

BP = blood pressure, AST = aspartate aminotransferase, ALT = alanine aminotransferase, ALP= alkaline phosphatase, hsCRP = high-sensitivity C-reactive protein, PTH = parathyroid hormone, TC = total cholesterol, TG = triglyceride
