# Supplementary figures and images for: Serum Gamma-Glutamyltransferase Levels Predict Clinical Outcomes in Hemodialysis Patients
Source: PLoS One. 2015 Sep 16;10(9):e0138159. doi: 10.1371/journal.pone.0138159 (PMC4573328; doi:10.1371/journal.pone.0138159)

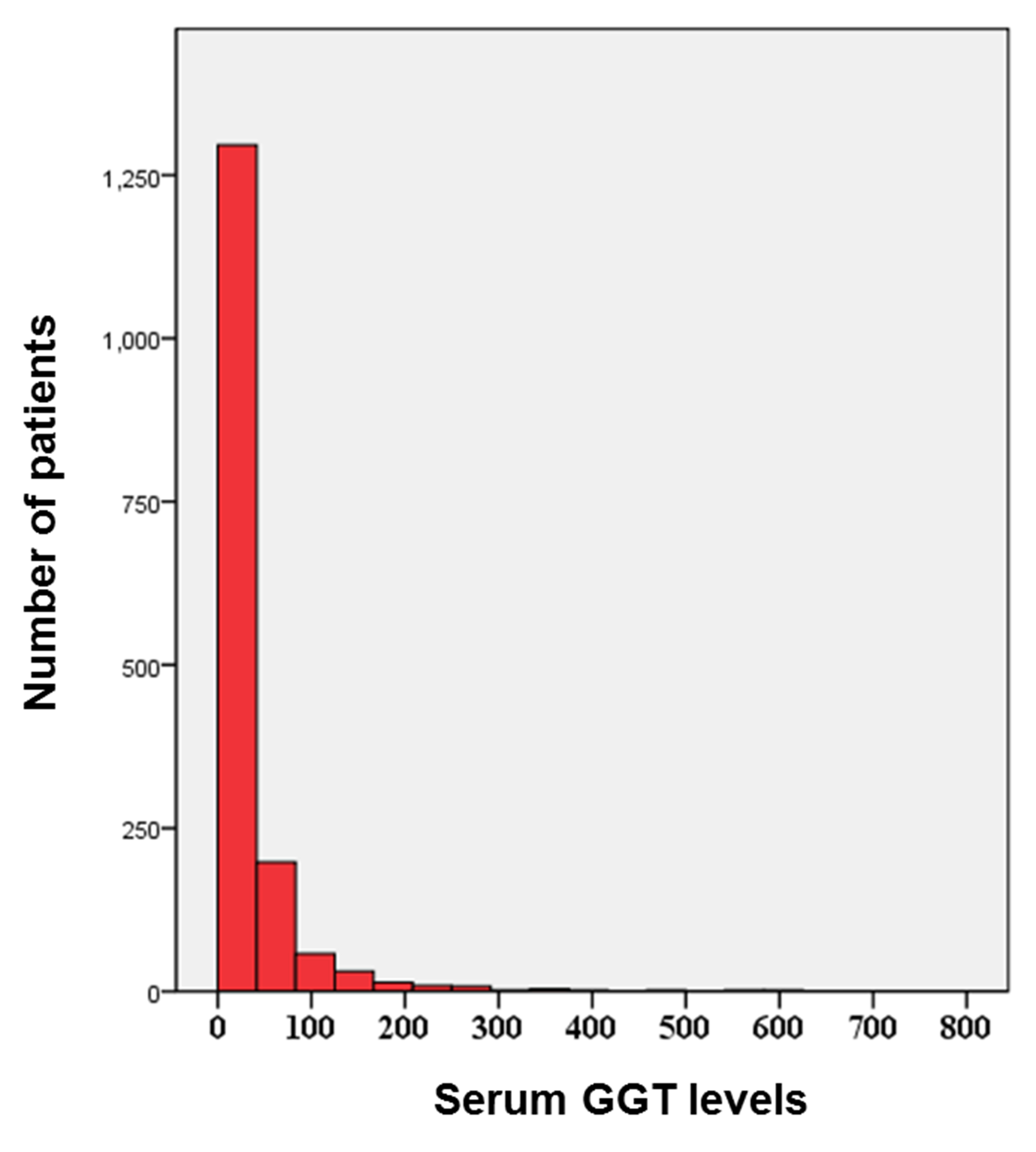

Supplement: S1 Fig — (TIF) [file pone.0138159.s002.tif]

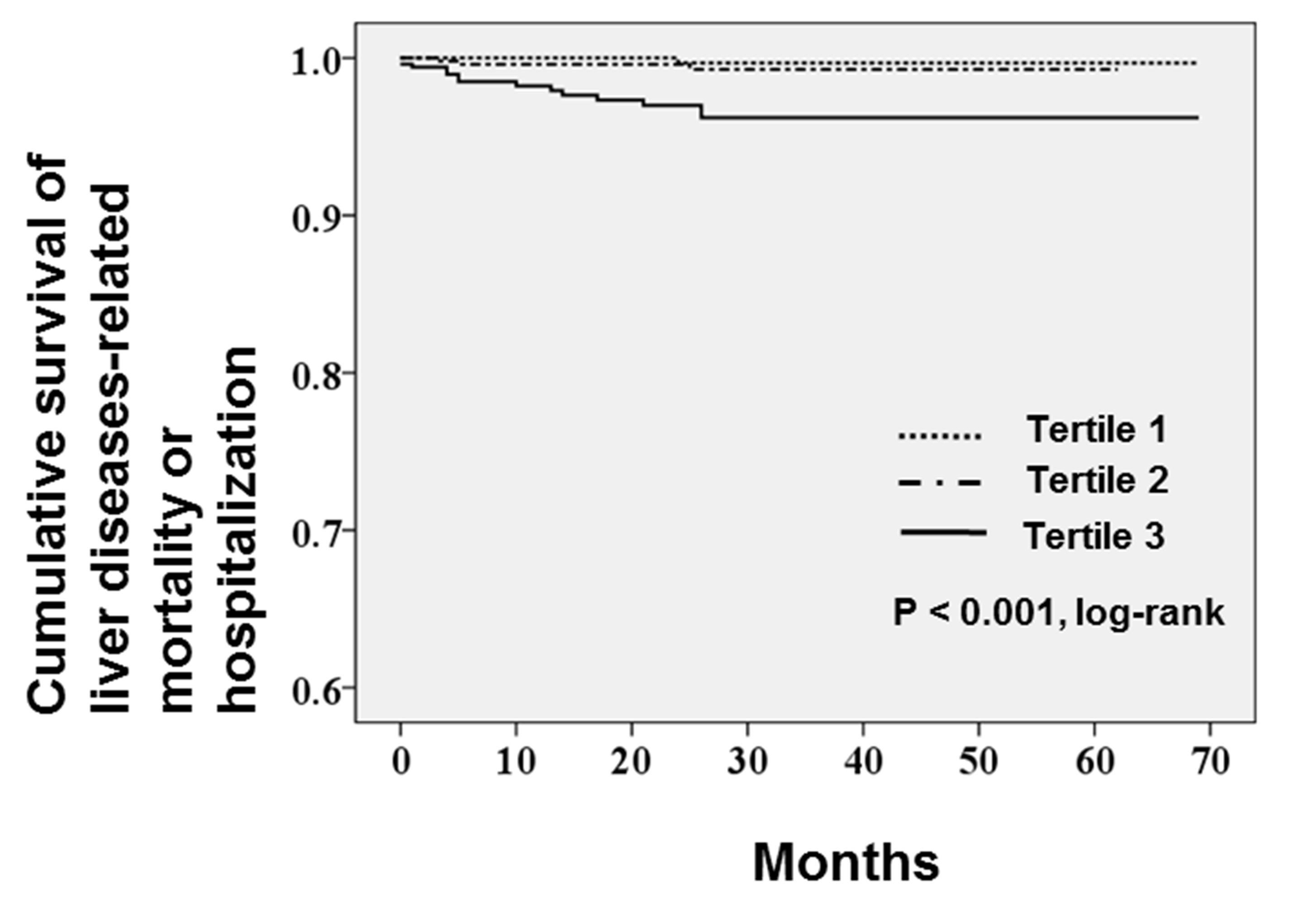

Supplement: S2 Fig — (TIF) [file pone.0138159.s003.tif]
